# Assessing LDL-C Levels and Lipid-Modifying Therapies in a Real-World Cohort of Patients with Atherosclerotic Cardiovascular Disease: The REALITY Study

**DOI:** 10.3390/jcm14072340

**Published:** 2025-03-28

**Authors:** Raquel Campuzano, José M. Mostaza, Vivencio Barrios, Isabel Egocheaga-Cabello, Jorge Francisco Gómez-Cerezo, Vicente Pallarés-Carratalá, Iciar Martínez-López, Mar Castellanos, Ignacio Hernández-Subirá, Nuria Morant-Talamante, Javier Parrondo, Luis Arigita-Lastra, José M. Gámez

**Affiliations:** 1 Department of Cardiology, Alcorcón Foundation University Hospital, 28922 Madrid, Spain; raquel.campuzano@salud.madrid.org; 2 Department of Internal Medicine, La Paz University Hospital, 28046 Madrid, Spain; 3 Department of Cardiology, Ramón y Cajal University Hospital, 28034 Madrid, Spain; 4 Family Medicine Service, Centro de Salud Isla de Oza, 28035 Madrid, Spain; 5 Department of Internal Medicine, Infanta Sofía University Hospital, 28702 Madrid, Spain; jfrancisco.gomez@salud.madrid.org; 6 Health Surveillance Unit, Unión de Mutuas, 12004 Castellón, Spain; pallares.vic@gmail.com; 7 Department of Medicine, Jaume I University, 12006 Castellón, Spain; 8 Pharmacy Department and Molecular Diagnostic and Clinical Genetics Unit, Son Espases University Hospital, 07120 Palma de Mallorca, Spain; 9 Department of Neurology, A Coruña University Hospital and Biomedical Research Institute, 15006 A Coruña, Spain; maria.del.mar.castellanos.rodrigo@sergas.es; 10 Smart Data Unit, Atrys Health, 28002 Madrid, Spain; 11 Medical Department, Novartis Pharmaceuticals, 08013 Barcelona, Spain; 12 Early Products and Health Economics Department, Novartis Pharmaceuticals, 08013 Barcelona, Spain; 13 Department of Cardiology, Son Llátzer University Hospital, 07198 Palma de Mallorca, Spain; 14 Departamento de Medicina, Universidad de las Islas Baleares, 07120 Palma de Mallorca, Spain; 15 CIBER de Fisiopatología de la Obesidad y la Nutrición (CIBEROBN CB 12/03/30038), Instituto de Salud Carlos III, 28029 Madrid, Spain

**Keywords:** atherosclerotic cardiovascular disease, real-world data, LDL-C levels, lipid-modifying therapies

## Abstract

**Background and Objectives:** Patients with atherosclerotic cardiovascular disease (ASCVD) remain at high risk of recurrent events. REALITY aims to analyse the actual levels of low-density lipoprotein cholesterol (LDL-C), the modalities of lipid-lowering therapies (LLT) used, and ASCVD patient outcomes in a real-world setting in Spain. **Methods:** REALITY is a retrospective observational study conducted using a healthcare database of 1.8 million patients representative of the Spanish population. The study included 26,976 patients with new or recurrent ASCVD events recruited from 2017 to 2019 and followed up for two years. **Results:** Management of ASCVD involved the utilisation of high-intensity (53%) or very high-intensity (36%) LLT. After two years, a decrease in total cholesterol (from 228.7 to 176.7 mg/dL), LDL-C (from 126.4 to 99.0 mg/dL), and triglycerides (from 216.7 to 163.7 mg/dL), accompanied by a moderate increase in HDL-C levels was observed. However, LDL-C goals of ESC/EAS guidelines were only reached by a minority of patients. Less than 15% of ASCVD patients achieved their LDL-C goals of <70, as stated in contemporary guidelines (3% if the present <55 mg/dL threshold in the ESC/EAS guidelines is considered). During the follow-up period, 9% of ASCVD patients died and 25% experienced a new ASCVD event. **Conclusions:** In the REALITY study, most patients did not achieve their target LDL-C goals despite receiving high- or very high-intensity LLT. Increasing the utilisation of extreme LLT is crucial to reducing recurrent ASCVD events and mitigating these patients’ high morbidity and mortality risk.

## 1. Introduction

Atherosclerotic cardiovascular disease (ASCVD) is the most frequent cause of cardiovascular disease (CVD) [1], which is responsible for over 45% of all deaths in Europe [2]. This condition also leads to high healthcare costs due to its significant use of resources [1]. However, up to 90% of CVD cases could be theoretically prevented by reducing several modifiable factors that influence CV risk, such as cigarette and alcohol consumption, blood pressure, cholesterol levels, diabetes, and excessive weight [3,4,5]. Hyperlipidaemia is one of the most relevant risk factors for ASCVD and a causal factor of atherosclerosis itself [6,7,8]. Extensive evidence from epidemiologic, genetic, and clinical intervention studies has shown that low-density lipoprotein (LDL) is causal in this process [9]. Thus, treating ASCVD requires reducing LDL-C levels by lipid-lowering treatments (LLTs) [10,11]. Several studies using LLTs indicate that the sooner and the lower LDL-C goals are achieved, the more significant the reduction in CV risk, with no adverse health effects observed so far in patients with deficient LDL-C levels [12,13,14].

Patients in secondary prevention are at a very high risk of experiencing another ASCVD event [4,5]. The European Society of Cardiology/European Atherosclerosis Society (ESC/EAS) 2016 Guidelines established a <70 mg/dL LDL-C goal for these patients [15]. Since 2019, ESC/EAS guidelines have recommended reducing at least 50% in LDL-C levels from baseline and an LDL-C goal of less than 55 mg/dL for patients at very high risk [4,5]. Despite these recommendations, the percentage of patients achieving their LDL-C goals in secondary prevention is low [16,17,18,19,20]. This may be due to therapeutic inertia caused by poor perception of the patient’s risk, low efficacy of LLT, lack of LLT combinations, and poor medication persistence/adherence [16,17,21,22,23]. However, although many recent studies have evaluated these aspects in experimental studies, there needs to be more real-life evidence on the degree of compliance with clinical guidelines. With the goal of better understanding LLT practices, REALITY studied the general characteristics, treatments used, and evolution of LDL-C levels in a real-world cohort of patients with ASCVD in Spain.

## 2. Materials and Methods

REALITY is a retrospective observational study conducted using the BIG-PAC^®^ healthcare database, which contains the anonymised medical records of 1.8 million patients from seven health regions in Spain (primary and hospital care, including all specialities), representing the overall Spanish population [24]. BIG-PAC^®^ covers all recorded information on diagnoses, tests, procedures, prescriptions, and treatments associated with each patient, with their corresponding dates. As detailed in the previously published REALITY protocol [25], the inclusion criteria were age ≥18 years; registry of an event or a first diagnosis of ASCVD during the recruitment period (1 January 2017 to 31 December 2018); being active in the database (≥2 records at least six months before the index date); and having ≥2 records during the follow-up period. In addition, patients should meet at least one of these three criteria: having a record of LDL-C ≥ 70 mg/dL (the objective of previous guidelines published in 2016 [15], which was the reference value during the recruitment period); being coded as diagnosis of hypercholesterolemia in the database registry; or being prescribed an LLT. Patients incorrectly identified in the database (e.g., birth date or sex have not been recorded) were not included in the study. The index date was defined as the date of the ASCVD event or the date of the first diagnosis during the recruitment period. After the index date, a two-year follow-up period was established (potentially lasting until the end of 2020 for the last recruited patients).

Patients were classified into subgroups according to their ASCVD-diagnosed pathology at the index date. This included two forms of coronary artery disease, angina and myocardial infarction (MI); two forms of cerebrovascular disease, ischaemic stroke and transient ischemic attack (TIA); and peripheral artery disease (PAD). The following variables were studied in the total ASCVD population and the study subgroups: age; sex; two-year incidence rates during the study period; lipid and anthropometric parameters; treatment duration and modifications; concomitant medication; new ASCVD events; mortality; and use of healthcare resources and costs. Recurrent ASCVD events were defined as a newly coded diagnosis of angina, MI, ischaemic stroke, TIA, aneurysm, embolism, or intermittent claudication. The calculation of the two-year % incidence rates, as defined in the study protocol [25], was estimated using the total number of different ASCVD cases recorded during two consecutive years (2017 and 2018) divided by the adult population included. Cut-off LDL-C values (55, 70, and 100 mg/dL) were used to stratify patients according to goal achievement [4,5]. The classification of LLT according to the intensity of the regimen was adapted from Escobar et al. [26], as depicted in Supplementary Table S1.

## 3. Results

### 3.1. Characteristics of the Study Population

**Figure 1:**
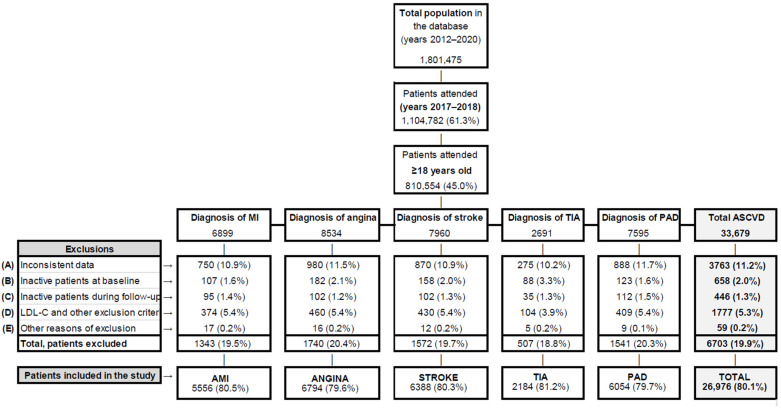
Flowchart depicting the number of patients recruited from the database according to the inclusion criteria. (A) Inconsistent data or partial loss of information within a patient’s records; (B) inactive patients in the database (<2 health records) for a minimum of 6 months before the study start; (C) inactive patients during follow-up (<2 health records during study period); (D) patients with a clinical report of LDL-C < 70 mg/dL, without a diagnosis of familial hypercholesterolemia, or without LLT treatment; (E) other reasons for exclusion due to incomplete data (e.g., no ID, year of birth, or sex recorded in the database).

From the total population in the database (1,801,475 patients), 1,104,782 (61.3%) had a record during the study period (Figure 1). From these, 810,554 (73.4%) were ≥18 years old (population used for the calculation of incidence). A total number of 33,679 subjects had an ASCVD event or a first diagnosis of ASCVD during the recruitment period (1 January 2017 to 31 December 2018). Of them, 26,976 (80.1%) met all the study inclusion criteria and were the patients included in subsequent analysis (Table 1).

Table 1 shows the patient distribution of the study population in the database according to their pathology subgroup and clinical situation, along with their sociodemographic characteristics and baseline comorbidities. Mean age was 69.9 ± 11.5 years, with a similar age range for all subgroups (Supplementary Table S2). Regarding sex distribution, 40.6% of patients were female, although this percentage was higher in some disease subgroups, such as TIA (51% females). The global incidence of new or recurrent ASCVD was 4.16%, 5.76% in men and 2.92% in women (Supplementary Table S3). This ratio, higher in men, was relatively similar in all disease subgroups, except for stroke, where a similar incidence was observed in both sexes.

### 3.2. Treatment Modalities at Baseline and During Follow-Up

Around 30% of the incident ASCVD population already received prior LLT at baseline, while the remaining cases (70%) started after the index event. Over 50% of patients with angina, stroke, TIA, or PAD as the index disease were on high-intensity LLT at the start of the follow-up period (Table 2). In contrast, MI patients were mainly treated with very high-intensity LLT regimens. During the two-year follow-up period, the percentage of patients receiving very high-intensity treatment increased from 36.4% to 37.1%, while extreme-intensity therapies changed from 0.9% to 1.2%. This increase is particularly evident in the MI group, in which 1.6% of patients received extreme-intensity therapy after the event; this percentage was 2.1% after two years.

### 3.3. Changes in LDL-C Levels and Other Lipid Parameters

**Figure 2:**
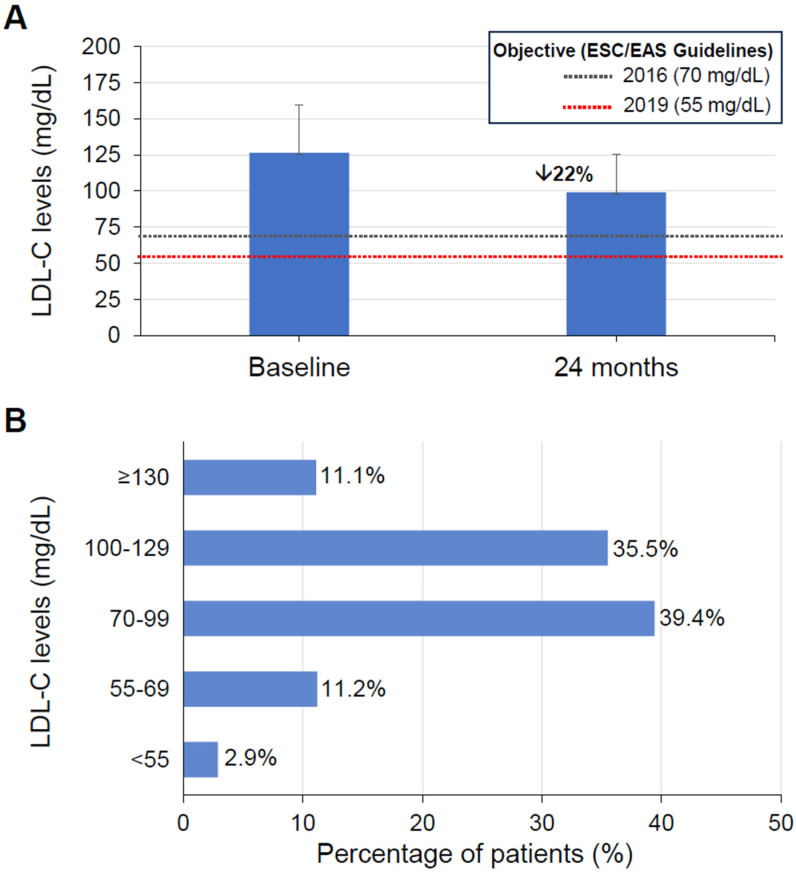
Degree of LDL-C control in ASCVD patients not previously receiving lipid-lowering therapies. (**A**) LDL-C (mg/dL) changes between baseline and at the two-year follow-up. Dashed lines indicate recommended goals by clinical guidelines [5,15]. Arrow points to the percentage reduction between baseline and follow-up. Error bars indicate standard deviation. (**B**) Distribution of ASCVD patients according to lipid control of LDL-C levels (mg/dL) at the two-year follow-up.

The mean baseline LDL-C level for the population was 126.4 ± 33.3 mg/dL. Patients receiving LLT before the index date (30% of the entire population) showed comparable LDL-C values at all time points: 103.9 ± 28.0 mg/dL at baseline, 103.0 ± 40.2 mg/dL after one year, and 94.9 ± 26.8 mg/dL after two years (Table 3). Patients not previously receiving LLT (70% of the entire population) had an average of 136.3 ± 36.1 mg/dL LDL-C at baseline; then, upon treatment administration, they reached 115.4 ± 38.9 and 100.9 ± 27.6 mg/dL LDL-C after one and two years of treatment, respectively. A 22% LDL-C reduction was observed between the baseline and the two-year follow-up point, reaching 99.0 ± 26.7 mg/dL levels (Figure 2A and Table 3), but reductions were not clearly observed in the first year; LLT prescriptions presented a constant reduction from index date, suggesting a reduction in treatment persistence (Supplementary Figure S2). However, mean values during the entire follow-up period remained well above target levels of current guidelines for very high risk (<55 mg/dL), although it should be noted that the recruitment period of the study (1 January 2017 to 31 December 2018) precedes the publication of ESC/EAS guidelines in 2019 [4]. At the two-year follow-up, 53.5%, 14.1%, and 2.9% of patients had LDL-C levels below 100 mg/dL, 70 mg/dL, and 55 mg/dL, respectively (Figure 2B). Changes in other lipid parameters are shown in Supplementary Table S4. Total cholesterol and triglycerides were reduced in all ASCVD subgroups, accompanied by a moderate increase in HDL-C levels.

### 3.4. Changes in Other CV Risk Factors

At baseline, ASCVD patients had systolic and diastolic blood pressure (BP) above recommended values (Supplementary Figure S1A,B). At the two-year follow-up, mean BP values fell to just below the recommended target values [5] (130 mmHg and 80 mmHg for systolic and diastolic BP, respectively). A reduction in HbA1c levels was also observed between baseline and the two-year follow-up (Supplementary Figure S1C), from 8% at baseline (the threshold of recommended values [5]) to 7.3%.

### 3.5. Morbidity and Mortality

**Figure 3:**
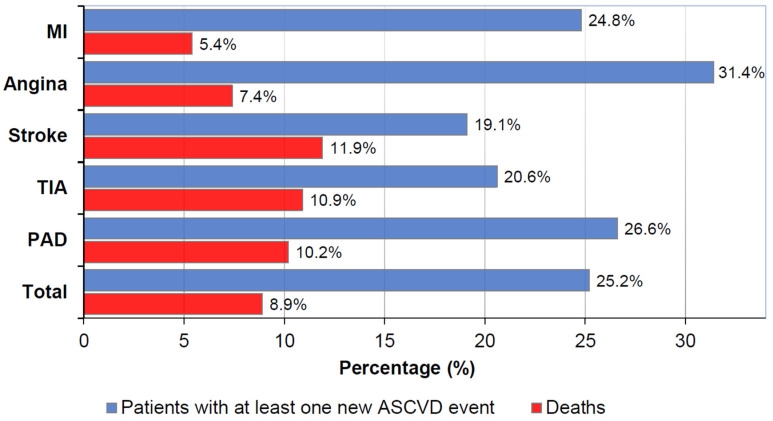
Percentage of patients who experienced at least one recurrent ASCVD event or died during the study period, according to the primary diagnosis. ASCVD events were defined as new coded diagnoses of angina, MI, ischaemic stroke, TIA, aneurysm, embolism, or intermittent claudication. ASCVD: atherosclerotic cardiovascular disease; MI: myocardial infarction; PAD: peripheral artery disease; TIA: transient ischaemic attack.

The mortality rate was 8.9%, with the highest rates recorded in the group on moderate-intensity treatments (11.1%). In comparison, the lowest rates were found in patients on extreme-intensity therapies (7.5%). Regarding morbidity, 25.2% of the population experienced a new ASCVD event. Nevertheless, there were several differences between each disease subgroup. The number of patients with new ASCVD events was higher in patients with angina as the baseline diagnosis (31.4%), followed by PAD (26.6%), MI (24.8%), TIA (20.6%), and stroke (19.1%) (Figure 3). From the recurrent cases, most new ASCVD events corresponded to the same event type recorded at the index date. However, the percentage of deaths followed a different pattern according to the primary diagnosis: stroke (11.9%), TIA (10.9%), PAD (10.2%), angina (7.4%), and MI (5.4%) (Supplementary Table S5).

## 4. Discussion

This real-world study provides evidence regarding the management of ASCVD in Spain, the characteristics of the patient population, the treatment modalities used, and the degree of LCL-C control. REALITY is the first real-life study to analyse separate data from all ASCVD disease subgroups, supporting that using LLT is not optimal in Spain [27]. The main strength of REALITY is the evaluation of real-world data using a database representative of the Spanish population [24]. The strength of the BIG-PAC^®^ database in generating reliable data for CV disease has been demonstrated previously [28], leading to solid conclusions that have been subsequently validated [29]. This allowed us to accurately estimate the demographic and clinical characteristics of patients with ASCVD. The database’s broad population of patients with ASCVD conferred a sufficient sample size to establish a sub-classification of patients according to their diagnosis at the index date. Moreover, REALITY provides an accurate and unbiased picture of treatment patterns in real clinical practice in Spain. This represents an advantage over previously cited case-control studies, registries, or cardiac rehabilitation programs [30], where healthcare professionals closely monitor patient treatment and follow-up.

The results of REALITY show that less than 15% of ASCVD patients achieved the goal of <70 mg/dL established by the 2016 guidelines in place during the recruitment period (1 January 2017 to 31 December 2018) [15]. In a very high-risk population, mostly treated with high- and very high lipid-lowering intensity treatments, less than 3% reached target LDL-C levels recommended for patients with high CV risk, currently <55 mg/dL according to the most recent 2019 ESC/EAS guidelines that were in place during the follow-up period of some of the patients of the study [4]. Even though the results of REALITY show a reasonable degree of control of several CV risk factors according to the 2021 ESC guidelines on CV disease prevention [5], such as BP and HbA1c, gaps in current lipid control strategies may exist between both healthcare settings. Lack of lipid control in an elevated number of patients on LLT not reaching LDL-C goals caused high morbidity and mortality in the ASCVD population, with over one in four patients experiencing a new ASCVD event and almost nine percent dying during the two years of the study. These results align with a robust body of evidence showing that increased LDL-C levels are closely related to recurrent ASCVD events and deaths [6,7,8]. These studies also showed that extreme/high-intensity LLT is associated with fewer events than moderate-intensity LLT.

The outcome of REALITY is consistent with studies performed in other regions, which also reported a lack of LDL-C control in ASCVD populations with very high CV risk [31,32,33,34,35]. Studies conducted in Europe also reported a low percentage of patients in secondary prevention meeting LDL-C goals [36]. In particular, the EUROASPIRE study [18] found that 71% and 37% of patients with a previous coronary event had LDL-C levels ≥70 mg/dL and ≥100 mg/dL, respectively. In contrast, the DA VINCI study confirmed that only 18% of patients on secondary prevention achieved LDL-C levels <55 mg/dL [19]. The SANTORINI study recruited patients at high and very high risk of CV from 14 European countries (including Spain) between 2020 and 2021, revealing that 80% failed to achieve the target LDL-C goals of the 2019 ESC/EAS guidelines [37]. A recent study in Spain, the Dyslipidemia Observatory study, analysed the impact of clinicians’ perception of the degree of LDL-C control, revealing that most patients do not achieve the recommended goals due to an incorrect perception of lipidic control, resulting in an insufficient intensification of LLT [38]. While the physician’s perception was that 62% of patients attained LDL-C goals (including those at high and very high risk of CV), the real data showed that this was the case for only 31% of patients.

With this line of evidence, REALITY calls for a profound change in the clinical management of LLT in Spain. Even though most patients on secondary prevention are currently treated with high- or very high-intensity LLT regimens, the particularly high-risk profile of the patients included in REALITY may present extra challenges in attaining therapeutic goals. Changes in LLT after the index date were intensified during follow-up for only a minority of patients despite dealing with a population that largely failed to reach LDL-C goals during this period. Even though the percentage of patients diagnosed with statin intolerance in the study population was low (5.3%) and this phenomenon is thought to be often overestimated [39], physicians may choose not to further intensify LLT due to fear of adverse events or lack of awareness of the crucial importance of achieving LDL-C goals [38]. Another physician-related factor may involve limited access to PCSK9 inhibitors. In Spain, ASCVD secondary prevention is shared between primary care and specialised healthcare professionals but can only be prescribed by the latter. Poor adherence is another factor that could explain the lack of lipid control [40]. In this sense, strategies to reduce the patient’s medication-related burden have been associated with improved adherence [41]. Lifestyle modifications have also been shown to improve LLT adherence [42] and could positively impact these studies’ contribution to prevention strategies and nonpharmacological approaches.

REALITY has some limitations intrinsic to retrospective studies using administrative databases. First, some information on the study population may be missing, such as the socioeconomic level of patients or attendance at specific cardiac rehabilitation programmes. Patients with angina and unstable angina cannot be distinguished in our study because this feature is only sometimes recorded in the database. Second, the data on some diseases may be incorrectly recorded due to possible inaccurate coding or variations in medical practice among professionals. Given that the events in the database are always associated with procedures, we may be underestimating the occurrence of pathologies that do not present acutely, such as angina and PAD. For example, the widely used ICD-9-CM coding system in Spain was reported to be inaccurate in identifying incident cases of stroke when not accompanied by physician notes or imaging studies to confirm valvular or vascular abnormalities [35,36]. Third, the inclusion criterion of patients with LDL > 70 mg/dL, diagnosis of hypercholesterolemia, or LLT prescription may have introduced a bias in the study since patients with a lack of lipidic control from baseline may be overrepresented. Fourth, the extent of LDL-C reduction during the follow-up may be mainly attributed to patients (70% of the total cohort) who were not receiving LLT before the index date, since they had their baseline treatment initiated immediately after their baseline LDL-C levels were recorded. We reached this conclusion because patients receiving LLT before the index date (30% of the total cohort) showed comparable LDL-C values at all points: close to 100 mg/dL already at baseline and in both follow-up time points. Therefore, the 22% LDL-C reduction observed on average is mainly explained by the initiation of treatment, as expected from the limited extent of treatment adjustments during the follow-up. Finally, since the follow-up period of REALITY lasted until the end of 2020, our results could be affected by the COVID-19 pandemic. Healthcare resources for non-COVID-19 pathologies were reduced during this period. In addition, mobility restrictions and fear of contracting COVID-19 infection at hospitals may have prevented ASCVD patients from attending healthcare services.

Future research should focus on several key areas to address the limitations identified in this study. Firstly, incorporating a broader set of data that includes socioeconomic information and patient participation in cardiac rehabilitation programs could provide a more comprehensive understanding of disease status and potential disparities in treatment efficacy. Increasing the use of electronic health records with detailed clinical notes could also mitigate inaccuracies in data recording. This, together with implementing more accurate and comprehensive coding systems, such as ICD-10-CM, would help ensure a precise identification of patient outcomes. Additionally, prospective study designs, rather than retrospective analyses, could reduce biases related to baseline lipid levels and improve data reliability by capturing real-time changes in patient management and outcomes. Furthermore, studies should evaluate the long-term effects of LLT intensification strategies and the impact of lifestyle modifications on LDL-C control, mortality, and new ASCVD events. Finally, research focusing on integrating multidisciplinary care teams could offer insights into optimising lipid management and overcoming healthcare delivery barriers, especially during pandemics or other healthcare disruptions.

## 5. Conclusions

To conclude, REALITY is the first study in Spain using a large sample of patients in a real-world setting to demonstrate that the degree of LDL-C reduction largely fails to meet target levels in a population at very high risk. Although the control of BP and HbA1c is being achieved, LDL-C goals of ESC/EAS guidelines are only reached by a minority of patients, yet very few treatment adjustments are made. The real-world outcome of low attainment of LDL-C goals was associated with high mortality and new events within two years of follow-up. Therefore, it is crucial to implement a new model that would ensure adequate treatment optimisation at all stages of care and promote bending the ASCVD curve by establishing a global change in lipid control management.

## Figures and Tables

**Table 1:** Sociodemographic characteristics and baseline comorbidities of ASCVD patients.

Index Event ^a^	MI	Angina	Stroke	TIA	PAD	Total
Number of patients, n (%)	5556 (20.6%)	6794 (25.2%)	6388 (23.7%)	2184 (8.1%)	6054 (22.4%)	26,976 (100%)
Days of follow-up, mean ± SD	555.6 ± 228.1	539.2 ± 230.0	561.6 ± 226.2	567.1 ± 224.8	552.4 ± 228.3	555.2 ± 227.5
Age, mean ± SD	68.5 ± 12.3	69.6 ± 11.5	71.2 ± 11.8	71.5 ± 11.2	69.6 ± 10.3	69.9 ± 11.5
Sex, n (% males)	3322 (59.8%)	4280 (63%)	3405 (53.3%)	1070 (49%)	3953 (65.3%)	16,030 (59.4%)
Patients with prior ASCVD events *, n (%)	1826 (32.9%)	1490 (21.9%)	1648 (25.8%)	522 (23.9%)	1666 (27.5%)	7152 (26.5%)
Comorbidities, n (%)						
Arterial hypertension	3556 (64%)	4273 (62.9%)	4267 (66.8%)	1437 (65.8%)	4111 (67.9%)	17,644 (65.4%)
Heart failure	833 (15%)	1141 (16.8%)	1035 (16.2%)	349 (16%)	1090 (18%)	4448 (16.5%)
Atrial fibrillation	783 (14.1%)	931 (13.7%)	1003 (15.7%)	293 (13.4%)	708 (11.7%)	3718 (13.8%)
Diabetes	1517 (27.3%)	2106 (31%)	2012 (31.5%)	681 (31.2%)	2028 (33.5%)	8344 (30.9%)
Obesity	1061 (19.1%)	1386 (20.4%)	1188 (18.6%)	391 (17.9%)	1193 (19.7%)	5219 (19.3%)
Active smoking	756 (13.6%)	707 (10.4%)	754 (11.8%)	236 (10.8%)	938 (15.5%)	3391 (12.6%)
Hypercholesterolaemia	3078 (55.4%)	3784 (55.7%)	3616 (56.6%)	1164 (53.3%)	3275 (54.1%)	14,917 (55.3%)
Hypertriglyceridaemia	1183 (21.3%)	1481 (21.8%)	1348 (21.1%)	439 (20.1%)	1241 (20.5%)	5692 (21.1%)
Mixed dyslipidaemia	800 (14.4%)	897 (13.2%)	952 (14.9%)	251 (11.5%)	739 (12.2%)	3639 (13.5%)
Fatty liver	728 (13.1%)	938 (13.8%)	824 (12.9%)	280 (12.8%)	842 (13.9%)	3612 (13.4%)
COPD	728 (13.1%)	876 (12.9%)	811 (12.7%)	232 (10.6%)	835 (13.8%)	3482 (12.9%)
Chronic kidney disease	706 (12.7%)	876 (12.9%)	843 (13.2%)	295 (13.5%)	957 (15.8%)	3677 (13.6%)
Depressive disorder	606 (10.9%)	985 (14.5%)	805 (12.6%)	251 (11.5%)	817 (13.5%)	3464 (12.8%)
Statin intolerance	233 (4.2%)	442 (6.5%)	332 (5.2%)	129 (5.9%)	309 (5.1%)	1445 (5.3%)

ASCVD: atherosclerotic cardiovascular disease; COPD: chronic obstructive pulmonary disease; MI: myocardial infarction; PAD: peripheral artery disease; TIA: transient ischemic attack. ^a^ According to database registry coding. * A patient might have more than one ASCVD event and in different territories.

**Table 2:** Number of ASCVD patients in each LLT regimen and medication switch during follow-up.

Index Event, *n*	MI *n* = 5556	Angina *n* = 6794	Stroke *n* = 6388	TIA *n* = 2184	PAD *n* = 6054	Total ASCVD *n* = 26,976
LLT started before the index date, *n* (%)	1464 (26.3%)	2190 (32.2%)	1974 (30.9%)	688 (31.5%)	1858 (30.7%)	8174 (30.3%)
Extreme intensity, *n* (%)						
After the event	89 (1.6%)	58 (0.9%)	19 (0.3%)	7 (0.3%)	61 (1%)	234 (0.9%)
One year	95 (1.7%)	65 (1%)	19 (0.3%)	7 (0.3%)	68 (1.1%)	302 (1.1%)
Two years	114 (2.1%)	76 (1.1%)	19 (0.3%)	8 (0.4%)	71 (1.2%)	337 (1.2%)
Very high intensity, *n* (%)						
After the event	2850 (51.3%)	2503 (36.8%)	2293 (35.9%)	605 (27.7%)	1580 (26.1%)	9831 (36.4%)
One year	2836 (51.0%)	2526 (37.2%)	2334 (36.5%)	618 (28.3%)	1573 (26.0%)	10,143 (37.6%)
Two years	2766 (49.8%)	2484 (36.6%)	2357 (36.9%)	622 (28.5%)	1515 (25.0%)	9997 (37.1%)
High intensity, *n* (%)						
After the event	2345 (42.2%)	3579 (52.7%)	3437 (53.8%)	1267 (58%)	3657 (60.4%)	14,285 (53.0%)
One year	2261 (40.7%)	3411 (50.2%)	3116 (48.8%)	1175 (53.8%)	3444 (56.9%)	13,610 (50.5%)
Two years	2048 (36.9%)	3064 (45.1%)	2395 (37.5%)	963 (44.1%)	2963 (48.9%)	11,625 (43.1%)
Moderate intensity, *n* (%)						
After the event	211 (3.8%)	491 (7.2%)	478 (7.5%)	238 (10.9%)	539 (8.9%)	1957 (7.3%)
One year	163 (2.9%)	374 (5.5%)	367 (5.7%)	191 (8.7%)	403 (6.7%)	1530 (5.7%)
Two years	130 (2.3%)	285 (4.2%)	278 (4.4%)	156 (7.1%)	313 (5.2%)	1186 (4.4%)
Non-statin, *n* (%)						
After the event	61 (1.1%)	163 (2.4%)	161 (2.5%)	67 (3.1%)	217 (3.6%)	669 (2.5%)
One year	59 (1.1%)	174 (2.6%)	148 (2.3%)	61 (2.8%)	256 (4.2%)	720 (2.7%)
Two years	58 (1.0%)	141 (2.1%)	177 (2.8%)	65 (3.0%)	270 (4.5%)	732 (2.7%)

ASCVD: atherosclerotic cardiovascular disease; MI: myocardial infarction; PAD: peripheral artery disease; TIA: transient ischaemic attack. Classification of LLT adapted from Escobar et al. [26] (see Supplementary Table S1).

**Table 3:** Changes in LDL-C levels (mg/dL) in ASCVD patients according to the presence or absence of LLT treatment before the index date.

Index Event	MI	Angina	Stroke	TIA	PAD	Total ASCVD
Number of patients, *n*	5556	6794	6388	2184	6054	26,976
Baseline, mean ± SD	**126.5 ± 33.5**	**127.0 ± 32.8**	**125.0 ± 32.7**	**127.6 ± 32.5**	**126.5 ± 34.4**	**126.4 ± 33.3**
Prior LLT	103.0 ± 27.6	104.1 ± 27.8	103.3 ± 27.4	105.2 ± 27.5	104.1 ± 29.8	103.9 ± 28.0
No prior LLT	134.9 ± 36.7	137.9 ± 35.5	134.8 ± 35.8	137.9 ± 35.4	136.3 ± 37.0	136.3 ± 36.1
One-year follow-up, mean ± SD	**110.2 ± 29.2**	**111.8 ± 29.1**	**110.4 ± 29.2**	**113.3 ± 29.2**	**112.2 ± 30.8**	**111.6 ± 29.5**
Prior LLT	101.9 ± 41.5	103.3 ± 38.8	102.4 ± 41	104 ± 39.4	103.3 ± 40.4	103.0 ± 40.2
No prior LLT	113.2 ± 38.3	115.9 ± 39.0	114 ± 40.2	117.6 ± 38.5	116.2 ± 38.7	115.4 ± 38.9
Two-year follow-up, mean ± SD	**97.7 ± 26.3**	**99.3 ± 26.3**	**98.3 ± 26.6**	**101.0 ± 26.4**	**99.9 ± 27.7**	**99.0 ± 26.7**
Prior LLT	94.3 ± 25.9	92.6 ± 25.3	94.8 ± 25.8	97.7 ± 26	95.8 ± 27.5	94.9 ± 26.8
No prior LLT	101.7 ± 27	99.6 ± 27.4	99.9 ± 27.5	102.5 ± 27.3	101.7 ± 28.5	100.9 ± 27.6

ASCVD events were defined as new coded diagnoses of angina, MI, ischaemic stroke, TIA, aneurysm, embolism, or intermittent claudication. ASCVD: atherosclerotic cardiovascular disease; LDL-C: low-density lipoprotein cholesterol; LLT: lipid-lowering treatment; MI: myocardial infarction; PAD: peripheral artery disease; SD: standard deviation; TIA: transient ischaemic attack.

## Data Availability

The data supporting this study’s findings are available from the corresponding author upon reasonable request.

## Supplementary Materials

The following supporting information can be downloaded at: https://www.mdpi.com/article/10.3390/jcm14072340/s1, Table S1: Classification of LLT modalities according to their intensity; Table S2: Distribution of ASCVD patients and lipid-lowering treatment by age range; Table S3: Estimation of two-year percentage Incidence rates of ASCVD patients in the database; Table S4: Changes in other lipid parameters (mg/dL) in ASCVD patients; Table S5: Number of deaths and new ASCVD events during the follow-up period; Figure S1: Changes in blood pressure (BP) and glycosylated haemoglobin (HbA1c) between baseline and at the two-year follow-up in patients with stablished ASCVD; Figure S2: Persistence of lipid-lowering therapies from index date during study follow up, based on prescriptions registered in the database.
