# Supplementary material for: Assessing LDL-C Levels and Lipid-Modifying Therapies in a Real-World Cohort of Patients with Atherosclerotic Cardiovascular Disease: The REALITY Study
Source: J Clin Med. 2025 Mar 28;14(7):2340. doi: 10.3390/jcm14072340 (PMC11989470; doi:10.3390/jcm14072340)
Supplement: Supplementary file 1 [file jcm-14-02340-s001.zip › jcm-3527247-supplementary.pdf]

SUPPLEMENTARY MATERIAL

Table S1. Classification of LLT modalities according to their intensity.

Lowering ability	Treatments
**Extreme (76-85% reduction)**	**Add PCSK9 inhibitor at maximally tolerated doses to LLT**<br>Evolocumab 140 mg (~85%)<br>Alirocumab 75 mg (~76%)<br>Alirocumab 150 mg
**Very high (60-75% reduction)**	**High-potency statin + ezetimibe**<br>Atorvastatin 40-80 mg + ezetimibe 10 mg<br>Rosuvastatin 10-40 mg + ezetimibe 10 mg
**High (50-59% reduction)**	**High-potency statin**<br>Atorvastatin 40-80 mg<br>Rosuvastatin 20-40 mg<br>**Medium-potency statin + ezetimibe**<br>Atorvastatin 10-20 mg + ezetimibe 10 mg<br>Rosuvastatin 5 mg + ezetimibe 10 mg<br>Simvastatin 20-40 mg + ezetimibe 10 mg<br>Pravastatin 40 mg + ezetimibe 10 mg<br>Lovastatin 40 mg + ezetimibe 10 mg<br>Pitavastatin 2-4 mg + ezetimibe 10 mg<br>Fluvastatin XL 80 mg + ezetimibe 10 mg
**Moderate (30-49% reduction)**	**Medium-potency statin**<br>Atorvastatin 10-20 mg<br>Rosuvastatin 5-10 mg<br>Simvastatin 20-40 mg<br>Pravastatin 40 mg<br>Lovastatin 40 mg<br>Pitavastatin 2-4 mg<br>Fluvastatin XL 80 mg<br>**Low potency statin + ezetimibe**<br>Simvastatin 10 mg + ezetimibe 10 mg<br>Pravastatin 20 mg + ezetimibe 10 mg<br>Lovastatin 20 mg + ezetimibe 10 mg<br>Fluvastatin 40 mg + ezetimibe 10 mg<br>Pitavastatin 1 mg + ezetimibe 10 mg

Low potency statins + ezetimibe are considered to cause moderate reductions (30-49%), medium potency statins + ezetimibe are considered to cause high decreases (50-59%), and high potency statins + ezetimibe are deemed to cause very high reductions (60-75%). Source: Escobar et al. Recommendations to improve lipid control. Consensus document of the Spanish Society of Cardiology. Rev Esp Cardiol (Engl Ed). 2020;73(2):161-167 [26]. LLT: lipid-lowering treatment.

Table S2. Distribution of ASCVD patients and lipid-lowering treatment by age range.

A. **Distribution of ASCVD patients by age range**						
**Groups, *n***	**MI** 5,556	**Angina** 6,794	**Stroke** 6,388	**TIA** 2,184	**PAD** 6,054	**Total** 26,976
**18-44 years, n (%)**	128 (2.3%)	143 (2.1%)	114 (1.8%)	39 (1.8%)	79 (1.3%)	503 (1.9%)
**45-64 years, n (%)**	2117 (38.1%)	2167 (31.9%)	1738 (27.2%)	535 (24.5%)	1828 (30.2%)	8385 (31.1%)
**65-74 years, n (%)**	1606 (28.9%)	2140 (31.5%)	1872 (29.3%)	660 (30.2%)	2204 (36.4%)	8482 (31.4%)
**≥ 75 years, n (%)**	1705 (30.7%)	2344 (34.5%)	2664 (41.7%)	950 (43.5%)	1943 (32.1%)	9606 (35.6%)

B. **Distribution of lipid-lowering treatment by age range and sex**				
**Cohorts of study**	**Extreme**	**Very high**	**High**	**Moderate**
**Age (years)**				
Mean (SD)	66.1 (11.4)	67.9 (11.3)	70.9 (11.4)	73.9 (11)
Median (P25-P75)	67.6 (60.1 - 73.6)	68.4 (59.7 - 76.4)	71.5 (63.3 - 79.7)	74.7 (67.1 - 82.3)
Min-Max	33.8 - 87.8	24.6 - 98.1	21.3 - 98.1	26.8 - 98.6
**Age ranges (N, %)**				
18 - 44 years, n (%)	16 (6.7%)	230 (2.3%)	218 (1.6%)	14 (0.7%)
45 - 64 years, n (%)	90 (37.5%)	3706 (37.3%)	3968 (27.9%)	378 (19.4%)
65 - 74 years, n (%)	86 (35.8%)	3094 (31.2%)	4530 (31.9%)	596 (30.6%)
≥75 years, n (%)	48 (20%)	2900 (29.2%)	5488 (38.6%)	962 (49.3%)
**Sex (males)**	168 (70%)	6458 (65%)	8014 (56.4%)	994 (51%)

ASCVD: atherosclerotic cardiovascular disease; MI: myocardial infarction; PAD: peripheral artery disease; TIA: transient ischaemic attack.

Table S3. Estimation of two-year percentage Incidence rates of ASCVD patients in the database.

Diagnosis	Number of cases*	Population	% Incidence [95% CI]
MI			
**Men**	5,178	352,591	1.47% [1.43 - 1.51]
**Women**	2,574	457,963	0.56% [0.54 - 0.58]
**Total**	**7,752**	**810,554**	**0.96% [0.94 - 0.98]**
Angina			
**Men**	6,243	352,591	1.77% [1.73 - 1.81]
**Women**	3,634	457,963	0.79% [0.77 - 0.82]
**Total**	**9,877**	**810,554**	**1.22% [1.19 - 1.24]**
Stroke			
**Men**	4,476	352,591	1.27% [1.23 - 1.31]
**Women**	4,486	457,963	0.98% [0.95 - 1.01]
**Total**	**8,962**	**810,554**	**1.11% [1.08 - 1.13]**
TIA			
**Men**	1,980	352,591	0.56% [0.54 - 0.59]
**Women**	1,187	457,963	0.26% [0.24 - 0.27]
**Total**	**3,167**	**810,554**	**0.39% [0.38 - 0.4]**
PAD			
**Men**	5,416	352,591	1.54% [1.5 - 1.58]
**Women**	3,419	457,963	0.75% [0.72 - 0.77]
**Total**	**8,835**	**810,554**	**1.09% [1.07 - 1.11]**
Total ASCVD			
**Men**	20,311	352,591	5.76% [5.68 - 5.84]
**Women**	13,368	457,963	2.92% [2.87 - 2.97]
**Total**	**33,679**	**810,554**	**4.16% [4.11 - 4.2]**

ASCVD: atherosclerotic cardiovascular disease; CI: confidence interval; IR: incidence rate; MI: myocardial infarction; ND: undetermined; PAD: peripheral arterial disease; TIA: transient ischaemic attack. *A patient might have more than one ASCVD event in different territories.

Table S4. Changes in other lipid parameters (mg/dL) in ASCVD patients.

Groups, *n*		MI<br>5,556	Angina<br>6,794	Stroke<br>6,388	TIA<br>2,184	PAD<br>6,054	Total<br>26,976
**Total cholesterol, mean ± SD**	**Baseline**	231.5 ± 45.6	229.0 ± 44.2	225.7 ± 44.1	229.6 ± 41.7	228.8 ± 44.8	228.7 ± 44.4
	**2 years**	176 ± 36.3	176.6 ± 35.8	174.9 ± 36.9	179.3 ± 34.4	178.3 ± 37.0	176.7 ± 36.3
**HDL-C, mean ± SD**	**Baseline**	46.5 ± 11.0	47.6 ± 11.1	48.2 ± 11.6	49.2 ± 11.7	47.8 ± 12.0	47.7 ± 11.5
	**2 years**	55.7 ± 11.2	56.2 ± 11.1	56.6 ± 11.5	57.3 ± 11.7	55.9 ± 11.9	56.2 ± 11.4
**Triglycerides, mean ± SD**	**Baseline**	220.4 ± 77.2	217.5 ± 85.0	214.3 ± 68.9	212.8 ± 62.5	216.2 ± 87.9	216.7 ± 78.9
	**2 years**	167.2 ± 77.0	164.6 ± 84.7	161.4 ± 68.4	160.1 ± 62.4	163.2 ± 87.1	163.7 ± 78.5

ASCVD: atherosclerotic cardiovascular disease; HDL-C: high-density lipoprotein cholesterol; MI: myocardial infarction; PAD: peripheral artery disease; TIA: transient ischaemic attack.

Table S5. Number of deaths and new ASCVD events during the follow-up period.

Index event	MI	Angina	Stroke	TIA	PAD	Total
**Number of patients**	5,556	6,794	6,388	2,184	6,054	26,976
**Deaths, *n* (%)**	298 (5.4%)	500 (7.4%)	760 (11.9%)	238 (10.9%)	618 (10.2%)	2414 (8.9%)
**Number of patients with new events, *n* (%)**						
**One event**	1,356 (24.4%)	2,086 (30.7%)	1,188 (18.6%)	439 (20.1%)	1,550 (25.6%)	6636 (24.6%)
**More than one event**	22 (0.4%)	48 (0.7%)	32 (0.5%)	11 (0.5%)	61 (1.0%)	162 (0.6%)
**Number of new events**						
**Total events, n**	1,400	2,180	1,250	460	1,676	6,966
**MI events, n (%)**	657 (46.9%)	629 (28.9%)	26 (2.1%)	26 (5.7%)	46 (2.7%)	1,384 (19.9%)
**Angina events, n (%)**	409 (29.2%)	1,047 (48.0%)	148 (11.8%)	70 (15.2%)	248 (14.8%)	1,922 (27.6%)
**Stroke events, n (%)**	129 (9.2%)	213 (9.8%)	748 (59.8%)	95 (20.7%)	74 (4.4%)	1,259 (18.1%)
**TIA events, n (%)**	21 (1.5%)	35 (1.6%)	80 (6.4%)	177 (38.5%)	8 (0.5%)	321 (4.6%)
**PAD events, n (%)**	184 (13.1%)	256 (11.7%)	248 (19.8%)	92 (20.0%)	1300 (77.6%)	2,080 (29.9%)

ASCVD events were defined as new coded diagnoses of angina, MI, ischaemic stroke, TIA, aneurysm, embolism, or intermittent claudication. ASCVD: atherosclerotic cardiovascular disease; MI: myocardial infarction; PAD: peripheral artery disease; TIA: transient ischaemic attack.

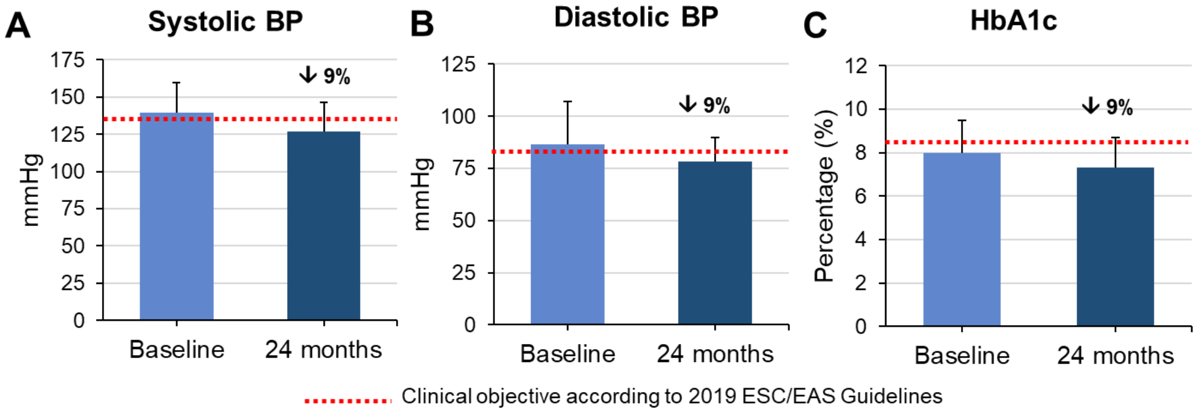

Figure S1. Changes in blood pressure (BP) and glycosylated haemoglobin (HbA1c) between baseline and at the two-year follow-up in patients with stablished ASCVD. **(A)** systolic BP; **(B)** diastolic BP; **(C)** HbA1c; Dashed lines indicate targets recommended by clinical guidelines [5]. Arrows point to the percentage reduction between baseline and follow-up. Error bars indicate standard deviation.

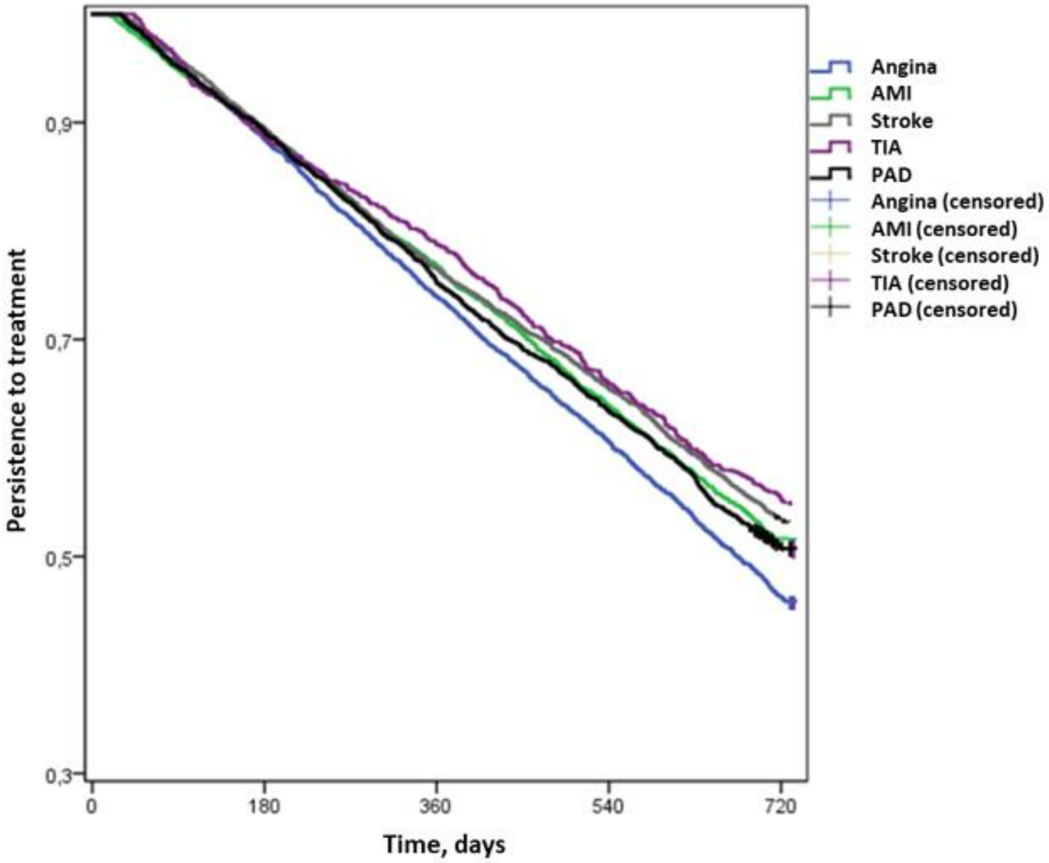

Figure S2. Persistence of lipid-lowering therapies from index date during study follow up, based on prescriptions registered in the database. AMI, acute myocardial infarction; PAD, peripheral artery disease; TIA, transient ischemic attack
